# Native Study of the Behaviour of Magnetite Nanoparticles for Hyperthermia Treatment during the Initial Moments of Intravenous Administration

**DOI:** 10.3390/pharmaceutics14122810

**Published:** 2022-12-15

**Authors:** Valentina Marassi, Ilaria Zanoni, Simona Ortelli, Stefano Giordani, Pierluigi Reschiglian, Barbara Roda, Andrea Zattoni, Costanza Ravagli, Laura Cappiello, Giovanni Baldi, Anna L. Costa, Magda Blosi

**Affiliations:** 1 Department of Chemistry G. Ciamician, University of Bologna, Via Selmi 2, 40126 Bologna, Italy; 2 Stem Sel srl, University of Bologna, 40129 Bologna, Italy; 3 CNR-ISSMC, Institute of Science, Technology and Sustainability for Ceramics (Former ISTEC), Via Granarolo 64, 48018 Faenza, Italy; 4 Ce.Ri.Col, Colorobbia Consulting S.R.L., 50059 Sovigliana Vinci, Italy

**Keywords:** biological identity, biological fluids, flow field flow fractionation (FFF)-multidetection, hyperthermia treatment, intravenous administration, magnetic nanoparticles, native characterization, protein corona

## Abstract

Magnetic nanoparticles (MNPs) present outstanding properties making them suitable as therapeutic agents for hyperthermia treatments. Since the main safety concerns of MNPs are represented by their inherent instability in a biological medium, strategies to both achieve long-term stability and monitor hazardous MNP degradation are needed. We combined a dynamic approach relying on flow field flow fractionation (FFF)-multidetection with conventional techniques to explore frame-by-frame changes of MNPs injected in simulated biological medium, hypothesize the interaction mechanism they are subject to when surrounded by a saline, protein-rich environment, and understand their behaviour at the most critical point of intravenous administration. In the first moments of MNPs administration in the patient, MNPs change their surrounding from a favorable to an unfavorable medium, i.e., a complex biological fluid such as blood; the particles evolve from a synthetic identity to a biological identity, a transition that needs to be carefully monitored. The dynamic approach presented herein represents an optimal alternative to conventional batch techniques that can monitor only size, shape, surface charge, and aggregation phenomena as an averaged information, given that they cannot resolve different populations present in the sample and cannot give accurate information about the evolution or temporary instability of MNPs. The designed FFF method equipped with a multidetection system enabled the separation of the particle populations providing selective information on their morphological evolution and on nanoparticle–proteins interaction in the very first steps of infusion. Results showed that in a dynamic biological setting and following interaction with serum albumin, PP-MNPs retain their colloidal properties, supporting their safety profile for intravenous administration.

## 1. Introduction

Nanoparticles (NPs) and nanomaterials (NMs) have been a focus of the biomedical sciences and engineering for over a century because of their enormous potential in nanotechnologies. Magnetic nanoparticles represent one of the most investigated class of nanomaterials for biomedical application due to their unique physicochemical properties that make them suitable for many applications in biotechnology, magnetic separation, targeted drug delivery, diagnostics (MRI, CT, PET, ultrasound, SERS), optical imaging, and as cytotoxic agents [1]. Furthermore, magnetic nanoparticles can be synthetized and modified with various chemical functional groups, which enables their conjugation with antibodies, ligands, and drugs of interest, thus opening their application for theragnostic purposes in cancer treatment, combining diagnosis with therapy [2,3,4]. Iron oxides particles, such as magnetite (Fe_3_O_4_) and its oxidized form (α-Fe_2_O_4_), are some of the most used magnetic carriers in biomedical applications. They have good biocompatibility, low toxicity [5], and are protagonists of one of the most promising cancer therapies: magneto fluid hyperthermia (MFH), which uses magnetic NPs to heat biological tissues and destroy cancer cells. In fact, cancer cells are more sensitive to high temperatures than healthy cells and die of apoptosis above a temperature of 43–46 °C [6]. The treatment can be used both alone or paired with other treatments such as chemotherapy and radiotherapy to optimize its efficiency. Fe_3_O_4_ magnetic nanoparticles (MNPs) should have a narrow size distribution and good dispersibility in aqueous media to be relevant for the human body. The narrow size distribution of the iron oxide core ensures high heating capabilities in a low concentration under biocompatible alternating magnetic field (AMF) conditions [7].

The main issues of these particles are represented by their loss of magnetism under oxidation and long-term inherent instability due to the high surface energy and the strong magnetic attraction between particles. High salt concentrations typical of biological matrixes further affect the colloidal stability of MNPs [8]. These two main instability routes can be handled by surface functionalization. Several classes of biocompatible chelating agents (lipids, gelatin, dextran, chitosan, polyvinyl alcohol, etc.) are nowadays used to form a polymeric layer on the surface of magnetic NPs to improve their stabilization considering their biological application [9,10]. Among various coatings, PEG—polyethylene glycol—stands out because of its hydrophilicity, biocompatibility, non-antigenicity, and antifouling properties [11]. In fact, PEG-coated iron oxide NPs have shown good results which remarkably extend NPs circulation in the blood [12]. Another interesting coating used on iron oxide NPs is the biodegradable copolymer PLGA, widely used for the preparation of biodegradable carriers and offering the possibility of tuning the drug release properties and the biological behaviour of Fe_3_O_4_ NPs [13,14,15].

Properly-coated MNPs, exploited for their biomedical applications, are typically dispersed in a biocompatible fluid and injected either directly into the tumor or in the blood system [16]. After entering the blood stream, they encounter serum proteins (i.e., human serum albumin, HSA, which makes up 60% protein content). As the infusion progresses, particles and proteins mix with an increasing imbalance towards the latter, generating a protein corona which likely allows MNPs to reach a new biological identity determined by this new biochemical surrounding. Corona effects are crucial for clinical applications since NPs properties and bioavailability are altered (in a positive or negative way) by their formation [17,18]. The process is a complex self-evolving scenario [19]; once MNPs in contact with the medium given the change in the medium MNPs encounter, an initial destabilization/precipitation/aggregation of MNPs, even with a subsequent restabilization, could occur and determine adverse reactions and potentially be very toxic for the patient.

Evaluating this transition proves challenging, since the interacting parts are numerous, and different parameters are needed to paint a full scenario. Studies focusing on the stability on MNPs in biological matrixes [20] and describing the formation and evolution of the protein corona [21] are typically performed with batch sizing and imaging tools [22]. These methodologies clarify whether NPs are subjected to modification but cannot give any answer other than an averaged one, without being able to identify the evolution of single populations [23]. Overall, this represents a major limitation, since the matrices involved are complex and the NPs samples are not always as monodispersed as presumed. A way to improve upon the limitations of these widespread methodologies is represented by the exploitation of Field Flow Fractionation (FFF) techniques. These techniques are a single phase-based platform which allow the separation of a wide range of different analytes (1 nm to tens of µm in size, corresponding to 15 orders of magnitude in molar mass) in native conditions based on their interactions with an external field [24,25]. A series of detectors coupled with the separation systems then provide a characterization of the separated analytes by the means of spectroscopy, laser scattering, and MS analysis. Different FFF subvariants can be described based on the external field exploited, and the Asymmetrical Flow FFF (AF4) variant represents by far the most exploited and successful of these [26]. AF4 has been widely used to separate and characterize systems of biomedical interest [27,28,29], biological matrices of high complexity [30,31], and to evaluate the binding interaction between different species [32,33]. Hollow Fiber Asymmetrical Flow FFF (HF5) represent the miniaturized version of AF4. Compared to AF4 and other typical particle-separation techniques, this variant typically has comparable or greater efficiency and higher sensitivity [34]. The separative channel is also disposable upon the need to avoid the risk of cross contamination. HF5 has recently been shown to characterize the behaviour of serum components in hemolysis conditions, allowing the discrimination of how each component separately interacts with heme in a competitive environment [35]. Moreover, its application on the characterization of metal NPs [36,37,38] and on their conjugation studies [39] is widely documented.

This paper reports the results collected in the framework of the EU project BIORIMA (H2020-760928_ BIOmaterial RIsk MAnagement), on Fe_3_O_4_ NPs coated by PEG/PLGA (PP-MNPs) intended for drug delivery application. PP-MNO were analysed by an HF5 platform at increasing amounts of HSA to simulate the first stages of intravenous administration and investigate their stability and behaviour in simulated use conditions. HF5 represents the best technique to monitor the morphological evolution of coated magnetic NPs applied by injection for therapy and subjected to a laminar flow. After characterizing PP-MNPs, we first monitored the size and zeta potential changes by a titration with HSA at four different points. Then, the same experiment was translated to a dynamic mode by exploiting a specifically designed HF5 multidetection method to observe the behaviour of PP-MNPs at a growing concentration of HSA, mimicking the first instants of administration. This platform provided a separation of the NPs-HSA conjugates from the leftover components and the simultaneous characterization of each separated species by the means of spectroscopy and laser scattering (MW and gyration radius). Moreover, the determination of the corresponding morphologies through the calculation of their v-values [40] provided us with outstanding information concerning the shape of the PP-MNPs and conjugates, the mechanisms with which HSA binds PP-MNPs, and helped address crucial gave us outstanding information concerning the shape of the PP-MNPs and conjugates, the mechanisms with which HSA binds PP-MNPs, and helped addressing address safety concerns.

## 2. Materials and Methods

### 2.1. Materials

Fe_3_O_4_ PEG-PLGA NPs (PP-MNP) were provided by Colorobbia Consulting S. r. l. (Sovigliana Vinci (FI), Italy) in the form of a suspension at a an Fe_3_O_4_ concentration of 2000 mg L^−1^ and prepared according to the patented procedure [41,42]. Briefly, MNPMNPs suspended in diethylene glycol were superficially functionalized with [N-(3,4-dihydroxyphenethyl) dodecanamide (DDA)] and dispersed in tetrahydrofuran (THF). Then, a THF solution of PLGA-b-PEG-COOH block copolymer was added to the magnetite-DDA NPs suspension. The formation of hybrid Fe_3_O_4_ PEG-PLGA was achieved by the nanoprecipitation method: two streams of fluid ((1) organic dispersion of functionalized magnetite and PLGA-b-PEG-COOH and (2) phosphate-buffered solution in a volumetric ratio of 1/10) were mixed and recovered. The formed dispersion was then dialyzed (Cogent M system, Pellicon membrane 2 Mini, cut-off 100 kDa) to remove the organic phase using a pure phosphate-buffered aqueous solution. The system was then concentrated to the final concentration and filtered through a polyethersulfone membrane syringe filter (0.22 mm). The overall process was carried out in sterile conditions. Human serum albumin (HSA) was purchased from Sigma-Aldrich (Milan, Italy).

### 2.2. Preparation of PP-MNP Stock Suspensions

The PP-MNP suspension, as provided, was diluted to 256 ppm in an aqueous solution containing 0.05%wt of filtered human serum albumin (HSA) by vortex treatment (30 s).

### 2.3. Characterization

#### 2.3.1. X-ray Diffraction (XRD)

XRD analysis was carried out on powder obtained by drying PP-MNP suspensions. The measurement was performed at room temperature with a Bragg/Brentano diffractometer (X’pertPro PANalytical) equipped with a fast X’Celerator detector, using a Cu anode as the X-ray source (Kα, λ = 1.5418 Å). Diffractogram was recorded in the range of 20–70° 2θ counting for 0.2 s every 0.05° 2θ step.

#### 2.3.2. Colloidal Characterization

The colloidal behaviour of PP-MNPMNPs was evaluated on stock and diluted stock suspensions in MilliQ water at 256 and 50 mg L^−1^ to determine the hydrodynamic size distribution and Zeta Potential (ZP), by means of dynamic light scattering (DLS) and electrophoresis light scattering (ELS) techniques, respectively. The measurements were performed using a Zetasizer Nanoseries (Malvern Instruments, Malvern, UK). Each sample was prepared and analysed in triplicate. Particle size diameter (d_DLS_) and zeta potential (Zpot_ELS_) values were obtained by averaging three measurements.

#### 2.3.3. HSA Titration

PP-MNP stock (2000 mg L^−1^) was diluted to 200 mg L^−1^. Four different batches of HSA were prepared in phosphate buffer to be mixed with magnetite to obtain an Fe_3_O_4_/HSA weight ratio of 2:1, 1:1, 1:2, and 1:4 as described in Table 1. The samples were prepared by mixing and homogenizing by vortex (30 s) the same volume of the two compounds (1 mL + 1 mL) at different concentrations.

The hydrodynamic diameter and Zeta Potential measurements were carried out in Phosphate Buffer (Sodium Phosphate 1 mM, pH 7.4) by the DLS/ELS technique.

#### 2.3.4. Transmission Electron Microscopy

PP-MNPs were observed by using an FEI TECNAI F20 microscope operating at 200 keV. The suspension was applied on a holey carbon-coated grid. The specimen was then dried at 60 °C. The images were collected in phase-contrast mode and high-angle annular dark-field scanning transmission mode (HAAD-FSTEM). High-resolution (HREM) and Selected Area Electron Diffraction (SAED) analyses were performed to investigate the crystalline phase structure and composition. The mean particle diameter was calculated on more than 100 particles.

#### 2.3.5. FFF UV FLD MALS

HF5 analyses were performed using an Agilent 1200 HPLC system (Agilent Technologies, Santa Clara, CA, USA) consisting of a degasser, an isocratic pump with an Agilent 1100 DAD UV/Vis spectrophotometer and an Agilent 1200 Fluorimeter combined with an Eclipse^®^ DUALTEC separation system (Wyatt Technology Europe, Dernbach, Germany), followed by an 18-angle multiangle light scattering detector model DAWN HELEOS (Wyatt Technology Corporation, Santa Barbara, CA, USA). The HF5 cartridge (Wyatt Technology, Europe) is commercially available and has a 10 kDa cutoff. The scheme of the HF5 cartridge, its assembly, and the modes of operation of the Eclipse^®^ DUALTEC system have already been described [43,44]. The ChemStation version B.04.02 (Agilent Technologies) data system for Agilent instrumentation was used to set and control the instrumentation and for the computation of various separation parameters, complete with the Wyatt Eclipse @ ChemStation version 3.5.02 (Wyatt Technology Europe). ASTRA^®^ software version 6.1.7 (Wyatt Technology Corporation) was used to handle signals from the detectors (MALS and UV) and to compute the sample Rg (radius of gyration), also named the RMS (root mean square radius) values. The HF5 method is composed of four steps: focus, focus–injection, elution, and elution–injection, allowing for flow equilibration, sample injection, sample separation and system cleaning. Longitudinal (transport) and transversal (focus/cross) flow settings are adjusted to customize the method. Longitudinal flow was kept constant at 0.35 mL/min, while cross/focus flow expressed as Vx are shown in Table 2. In normal fractionation mode, particle retention is a function of its apparent diffusion coefficient, relating retention time to hydrodynamic radius (*R*h). The Rh is approximated as a radius of a sphere having similar hydrodynamic behaviour in terms of diffusion and friction of the eluted particle [45]. Multi-angle laser light scattering (MALLS) was used to determine colloidal size. This technique allows for the determination of particle root mean square radius of gyration (Rg, or RMS) by measuring the net intensity of light scattered by such particles at a range of fixed angles. The particle Rg is determined by the mass distribution within the particle. The single mass increments are weighed by the square of the radius distance from the center of mass. Consequently, two particles with same hydrodynamic radius (Rh), but with different Rg values, may have a different mass distribution, and thus, different shapes [46].

The radius of gyration and molar mass distributions determined by FFF-MALS provide information on the scaling behaviours in the solution. The scaling exponent ν is defined by the slope in a double logarithmic logMW–logRg plot and gives information about the conformation of the molecules in the solution. It is theoretically defined for spheres ν = 0.33, random-coil ν = 0.5–0.6, and rod-like structures ν~1 [47,48].

## 3. Results and Discussion

### 3.1. Batch and Static Characterization

The first steps of the characterization of the evolution involved building a data set with the most common characterization techniques, which involved imaging, X-ray diffraction, and batch measurement of size (DLS) and zeta potential.

#### 3.1.1. X-ray Diffraction of MNP

**Figure 1:**
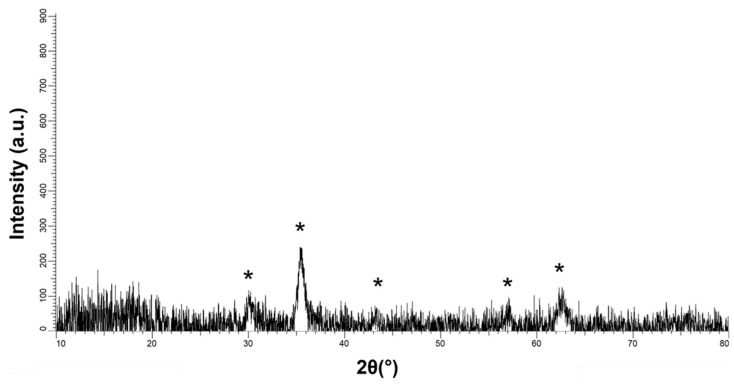
XRD diffractogram of MNPs (Fe_3_O_4_-PEG/PLGA). * = magnetite (JCPDS card n. 19-0629).

PP-MNPs were characterized through XRD. The main peaks identified on target NPs are consistent with the magnetite phase (JCPDS card n. 19-0629) with XRD reflections at 2Theta = 30.1°; 35.4°; 43.0°; 56.9°; and 62.5°, as shown by Figure 1.

The collected peaks were typically broad in agreement with the particle nanosized dimensions.

#### 3.1.2. Size and Zeta Potential Measurements

**Figure 2:**
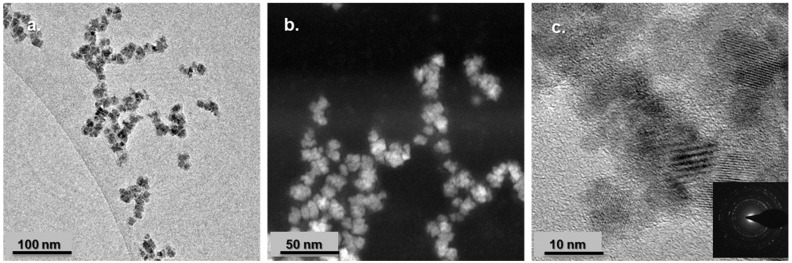
Transmission electron microscopy images of PP-MNPs (**a**) TEM phase-contrast image; (**b**) HAADF-STEM image; (**c**) HREM image and in the inset SAED polycrystalline pattern rings. Scale bars: (**a**) 100 nm; (**b**) 50 nm; (**c**) 10 nm.

PP-MNPs were observed by TEM. The images acquired in phase-contrast (Figure 2a) and HAADF-STEM (Figure 2b) modes revealed a regular spheroidal morphology of the particles with a mean diameter of 12 ± 4 nm. The higher magnification HREM image (Figure 2c) showed a cubic crystal structure consistent with the magnetite lattice; crystalline magnetite was the unique phase composition resulting from the SAED analysis of the collected polycrystalline pattern rings (inset of figure-c).

PP-MNPs dispersed in phosphate buffer and evaluated at two concentrations showed a neutral pH, a hydrodynamic diameter of about 70 nm, and a negative Z potential (~50 mV) as reported in Table 3. Results were consistent with previously-published works on PEG-PLGA coating [49,50].

As expected, the hydrodynamic diameter, comprehensive of the grafted polymers, was larger than the size assessed by TEM.

We also evaluated the colloidal properties of HSA at the highest and lowest concentrations used in dynamic-flow conditions, which as expected is present as predominantly a monomer in phosphate buffer with a size around 10 nm and a negative charge for both concentrations. It is interesting to note that for PP-MNPs, we obtained similar data at both concentrations, pointing out that the main colloidal properties were not affected by dilution and the change of stability, and the aggregation state is therefore due to the medium change following interactions in the biological setting.

#### 3.1.3. Titration with HSA in Static Conditions

**Figure 3:**
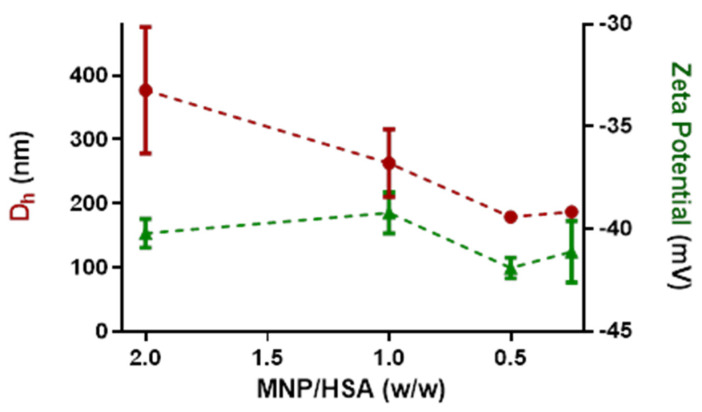
Titration of PP-MNPs with HSA in water. Red points: hydrodynamic diameter. Green triangles: zeta potential.

By means of DLS/ELS measurements, we monitored the colloidal evolution of magnetite nanoparticles titrated by HSA according to the mass ratios reported in Table 2. In our previous work [51], we reported that the addition of albumin increased the DLS diameters registered for the suspended nanoparticles. However, by titrating PP-MNPs with HSA in phosphate buffer, we observed a decrease in the hydrodynamic diameter for higher HSA amounts (Figure 3), consistent with a dispersion action or a size rearrangement promoted by the protein adsorption on nanoparticles.

From the DLS results, it is reasonable to infer that the increasing presence of HSA provides shielding and favors colloidal stability. For PP-MNPs with HSA ratios < 1, i.e., at predominant concentrations of HSA, the hydrodynamic diameter reached a plateau value around 180 nm (Table 4).

The PP-MNP-HSA suspensions showed Z potentials leveling off at around −40 mV, close to the MNPs value and slightly reduced for the presence of HSA, characterized by a lower Z potential.

Most importantly, the size deviation obtained for lower ratios (0.5 and 0.25, i.e., where HSA is more concentrated) is much lower, indicating that the structures obtained are more stable and defined.

### 3.2. Native and Dynamic Characterization with FFF-Multidetection

To simulate the intravenous injection administration, the titration experiment was translated from static conditions to an in-flow approach, using HF5 as separation device to isolate different populations, followed by online detection to monitor the profile changes in UV-Vis absorption, fluorescence, and measured size. PP-MNPs were characterized alone and in the presence of an increasing amount of HSA to understand their stability and behaviour once injected into a simulated biological medium. The points chosen, which were the same as those for the batch characterization, aimed to ideally photograph what occurs in the first moments of administration, when PP-MNPs shift from a scenario where they are the main species to one where the biological medium surrounds them. The analyses were carried out in phosphate buffer (Sodium Phosphate 1 mM, pH 7.4). The amount of PP-MNPs injected was the same for all analyses, and as shown in Table 2. All mixes were 1:1 in volume as to avoid changes due to different medium proportions.

The separation method was designed and optimized to elute HSA and PP-MNPs at different retention times. Method precision was assessed in triplicate on retention times and on signal intensity, which both exhibited deviations < 1%. The limit of quantification (LOQ) expressed as 10× the baseline noise resulted in 8.4 ng (0.42 pmol) and 48 ng for HSA and PP-MNPs, respectively. The final method envisioned a combination of gradient and isocratic crossflow which allowed for their baseline separation and elution in 30 min.

In this way, for the mixed suspensions, the insurgence of new bands or the coexistence of typical signals for the two species directly indicates an interaction between the two.

**Figure 4:**
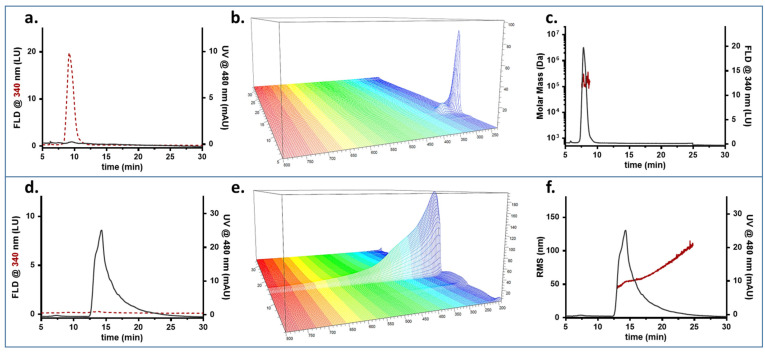
FFF fractogram (red dashed line: fluorescence signal; grey line: absorption signal), UV-Vis absorption spectrum, and Molar Mass/RMS radius (red) and fluorescence/UV profile (grey) obtained for HSA (**a**–**c**) and MNPs (**d**–**f**).

To obtain information about the composition of combined species, it was necessary to attribute a peak to one species, the other, or both, and “diagnostic” signals were selected. First, HSA displays intrinsic fluorescence (excitation at 280, emission at 340 nm), typical for proteins [48], while PP-MNPs only have a faint emission at 550 nm. Second, PP-MNPs absorb at 480 nm, while HSA does not. Thus, 480 nm was chosen to monitor the presence of PP-MNPs. The results are shown in Figure 4.

Figure 4a,d show the fractogram profiles recorded as absorption at 480 nm and fluorescence at 340 nm for the optimized method. As observed from the different profiles for the two signals and samples, the method developed ensured that HSA and PP-MNPs were potentially baseline-separated, and that both had a characteristic signal which could be followed individually.

HSA is eluted at 9 min as a single peak (Figure 4a) highlighting an absorption spectrum (Figure 4b) typical for a protein, with a local maximum at 280 nm and no absorption past the UV range. The molar mass averaged about 100 kDa (Figure 4c), indicating that HSA is present as a mix of monomer (66.7 kDa, prevalent) and oligomers as already observed in similar conditions with a native separation [serum heme].

PP-MNPs were eluted as a single band of a broad size distribution (red distribution, Figure 4f) peaking at 15 min (Figure 4d). The UV-Vis spectrum (Figure 4e) was broad and intense, with scattering at higher wavelengths. The population of NPs was found to be monomodal (Figure 4f)—the majority of PP-MNPs (min 14.0 to 17.5, 75% of peak area) had an Rg of 51 ± 5 nm while the peak tail reached 110 nm. This agrees with the DLS data measuring an Rh of 75 nm, since the shape factor obtained [52], expressed as the Rg/Rh ratio, would be equal to 0.7, corresponding to a solid sphere.

It is possible to see that the peak tailing contains aggregated forms of a higher radius, formed following contact with the saline environment.

**Figure 5:**
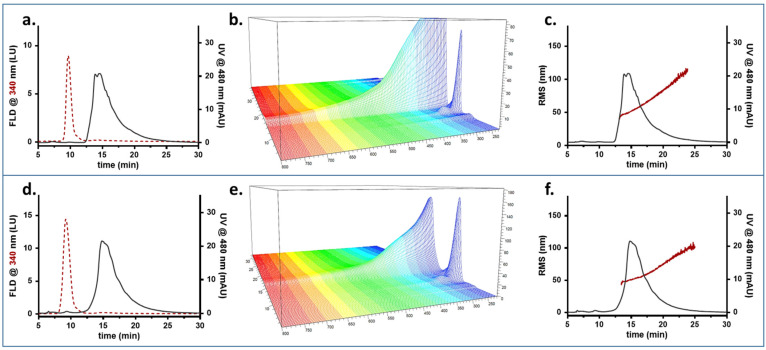
FFF fractogram (red dashed line: fluorescence signal; grey line: absorption signal), UV-Vis absorption spectrum, and Molar Mass/RMS radius (red) and fluorescence/UV profile (grey) obtained for suspensions at PP-MNP:HSA 2:1 (**a**–**c**) and 1:1 (**d**–**f**) mass ratios.

We then monitored the system evolution after mixing PP-MNPs with an increasing amount of has. Results referred to PP-MNP:HSA mixes at 2:1 and 1:1 mass ratios are shown in Figure 5. MALS measurements show the overlay of the RMS radius calculation on the signal at 480 nm used to visualize when NPs are eluted and their size/aggregation state. This signal allows the monitoring of the evolution in all mixtures. For each mix of PP-MNPs and HSA, the fractogram overlaying the HSA fluorescence and PP-MNPs absorption, the UV-Vis absorption spectrum, and the radius distribution are displayed.

In a 2:1 proportion, PP-MNPs and HSA are eluted at different times (Figure 5a, red dotted for HSA and grey line for MNP) and do not exhibit interactions apart from a slight absorption at 480 found at the HSA retention time, consisting of about 0.2% of the 480 signal-integrated area. The two different absorption spectra are shown by the 3D output (Figure 5b).

PP-MNPs are present as a single band of a broad size distribution similar to what is observed for PP-MNPs alone (Figure 5c). The largest part of the PP-MNPs is eluted from minute 14 to 17.5, corresponding to an average radius of 50 nm, while the peak tail reached a dimension of 110 nm (red distribution). For PP-MNPs and HSA mixed at a 1:1 ratio, we detected at 9 min a small peak at 480 nm (Figure 5d), meaning that some NPs are eluted earlier following conformational/surface modification. The absorption spectrum shows that the HSA profile is slightly different (absorbs at higher wavelengths as well, similar to the broad absorption of PP-MNPs), suggesting that some interactions take place between the two phases. In Figure 5d PP-MNPs are identified as a small band (more visible than the previous mixture) at 9 min (the intensity is however too low for a reliable RMS calculation) and a main monomodal band at 15 min with a broad size distribution as visible from the MALS radius calculation (Figure 5f). The majority of PP-MNPs (min 14.0 to 17.5) average 50 nm while the peak tail reaches 100 nm.

**Figure 6:**
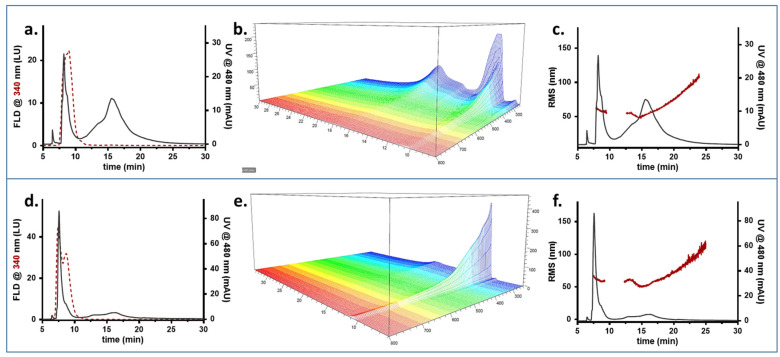
FFF fractogram (red dashed line: fluorescence signal; grey line: absorption signal), UV-Vis absorption spectrum, and Molar Mass/RMS radius (red) and fluorescence/UV profile (grey) obtained for 1:2 PP-MNP:HSA (**a**–**c**) and 1:4 PP-MNP:HSA (**d**–**f**) suspensions.

Data collected on the 1:2 PP-MNPs: HSA ratio, corresponding to a doubled HSA content, revealed a completely different situation, showing a clear interaction between the compounds (Figure 6).

The profile at 480 nm shows that nanoparticles from different species are eluted at different times. A new band absorbing at 8 min at 480 nm is clearly visible in Figure 6a, preceding the peak corresponding to HSA alone (Figure 6a), and the PP-MNP band at 14 min decreases the same amount since the total integrated area was verified to be constant for all analyses. Due to the fluorescence signal, we hypothesized that such new band also contains HSA, indicating that PP-MNPs and proteins interact to form a differently arranged system with a completely different retention behaviour and involving both species. The absorption spectrum appears as a combined band of HSA and PP-MNPs (Figure 6b). The MALS calculation pointed out that the newly-formed species had an RMS radius comparable to the previously characterized PP-MNPs (55 nm vs. 58 nm, Figure 6c). The early retention time for species with same RMS radius indicates a different shape and/or surface charge which impact the retentive behaviour in HF5. Since the DLS and Zeta potential results show a decreasing hydrodynamical radius, it is possible that HSA takes part in coating PP-MNPs competing with PEG/PLGA, the formal coating, and creates a hybrid coating less influenced by the polymer encaging. We hypothesize the formation of an HSA protein corona on PP-MNPs, with consequent formation of HSA-PP-MNPs composite. Last, the PP-MNPs: HSA 1:4 ratio showed a progression in the PP-MNPs shift to the earlier peak. The signal relative to HSA is split into a PP-MNP-containing fraction (eluted at 8 min) and its typical retention peak at 10 min, where the PP-MNPs also tailed (Figure 6d). The UV-Vis spectrum shows the migration of the absorption profile towards the earlier peak (Figure 6e); in terms of the RMS radius, the two species eluted at 8 and 15 min are measured at 60 nm and average at 59 nm, respectively (Figure 6f). A second shoulder of 60 nm at 12 min stems from the later band, suggesting a reconversion of the PP-MNPs towards the newly-formed band.

The fluorescence signal of HSA is split with a maximum at the same time of early-eluted PP-MNPs (whose peak increases compared to the 1:2 mix), and a second maximum at its characteristic retention time. The UV absorption shows that PP-MNPs are mainly present as an HSA-PP-MNPs composite (8 to 10 min) rather than particles alone (12 to 20 min). In the HSA excess condition (PP-MNPs: HSA ≥ 1:2), an HSA-PP-MNPs composite is formed. HSA gives rise to a protein corona on PP-MNPs, minimizing the agglomeration phenomena between PP-MNPs due to maximization of electrostatic and steric repulsion [53]. Thus, the HSA-PP-MNPs system was characterized by well-dispersed NPs and a net negative charge.

The FFF-multidetection data suggested that in the presence of a low amount of HSA, the two phases—HSA and PP-MNPs—coexist separately. As the HSA content increases, they begin to interact, and HSA becomes part of the existing PEG/PLGA coating, creating a protein corona on PP-MNPs, up to the encapsulation of most of the MNPs in the HSA matrix.

**Figure 7:**
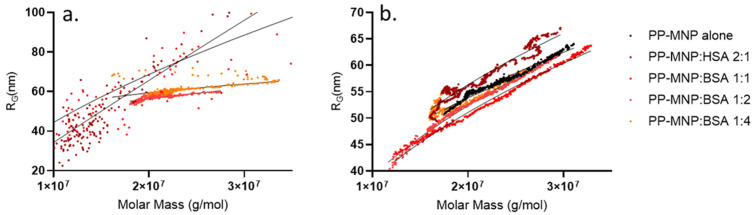
Conformation plots obtained for population 1 (**a**) and population 2 (**b**) expressed as double logarithmic molar mass/gyration radius regression lines.

Evidence of this transition also comes from the conformational study of the peaks of the HSA-PP-MNPs (7.5–10 min interval) and PP-MNPs (14–18 min interval), namely, population 1 and population 2, obtained for all mixes and shown in Figure 7.

A conformation plot considers the double logarithmic dependency between the mass and radius values calculated with MALS, where the slope “v” (v value) is an indication of the shape of the particles analysed. A solid sphere has a v value of about 0.33—higher values describe elongated or deformed structures (e.g., a rod has a v value of 1, whereas for a random coil v nears 0.7)—while lower values describe a denser core and a softer shell. In our case, population 2, i.e., free PP-MNPs still non-interacting with HSA, had a v value of 0.4 decreasing to 0.33 (0.40–0.41–0.40–0.36–0.33) at the highest ratio measured. These values indicate a spherical shape decreasing to a solid sphere at the lowering of the free PP-MNP amount, without evident conformational changes apart from what is expected from a lowering concentration. At the same time, when HSA increases, population 1 starts to form. In this case, the change in the conformation is very clear; it starts as a very elongated (and polydisperse) form (v value = 0.9 and 0.7 for 2:1 and 1:1 ratios), which can be attributed to HSA surrounding the PP-MNP system and solvating it. Then, the conformation plot evolves towards a core-shell form where dense PP-MNP particles are surrounded by a less-dense, stable coating of PEG/PLGA-HSA (v value = 0.21 and 0.19 for 1:2 and 1:4 ratios), which remained stable even when increasing the HSA:PP-MNP ratio.

This information is extremely relevant, since nanoparticle activity also depends on shape, surface, and conformation. Combined with the size results obtained from DLS, these data confirm that MNPs retain a spherical shape, and the change in retention time previously noted is not due to a new conformation, but rather to a different apparent radius. In fact, the retention time of the observed species is not compatible with the radius measured by MALS—the explanation for such little retention can be due to a reversed elution mode, meaning that the species is behaving as hydrodynamically very big while actually being smaller, which is only possible when species such as PEG surround it. Weak interactions between particles and free PEG/PLGA could contribute to creating a high apparent hydrodynamic radius which is eluted in inversed mode. The evidence for surface interactions occurring with the increase in HSA (as opposed to simple coelution) can be gathered also by observing a change in the emission spectra of the conjugate, similar to that of HSA (whereas PP-MNPs do not emit, see Figure 4d) but presenting additional peaks (not shown). The interaction can be due to electrostatic interactions between MNPs and the negative charge of HSA in a physiological pH (due to a pI of 4.7), but also on the chemisorption on the thiol groups from albumin onto the MNP surface. Moreover, the presence of a complex structure involving both PEG/PLGA and HSA over MNPs is confirmed by the absorption spectrum which is typical for PP-MNPs. As for PEG/PLGA behaviour, it is true that the protein-repellent properties of coatings such as PEG are reported to prevent nonspecific interactions of serum proteins with nanoparticles, reducing the interaction with the immune system and leading to longer circulation times in the bloodstream [54]. However, there are also some reports that BSA was able to rapidly couple to PEG-encapsulated nanoparticles to increase their stability and biocompatibility in the cell culture media and intracellularly by preventing aggregation [55,56], corroborating the mechanism hypothesized above.

**Figure 8:**
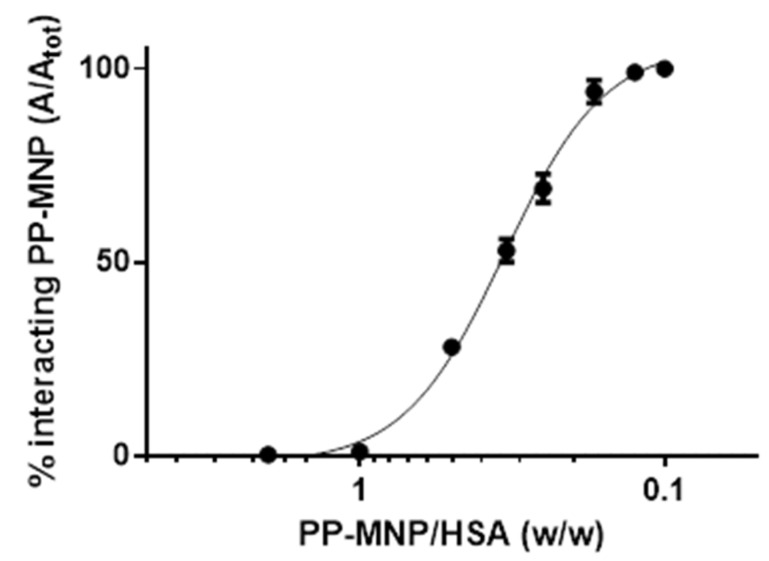
HSA/PP-MNP interaction vs. HSA increase expressed as the percentage of PP-MNPs interacting with HSA (black dots and error bars, n = 3) calculated from independent runs of supensions at 2:1, 1:1, 1:2, 1:4, 1:6, 1:8, 1:10 PP-MNPs:HSA mass ratio.

Last, to summarize our findings, we evaluated the area of the signal at 480 nm for all mixtures. The total area values were identical (within a 3% deviation), showing that the increase in HSA did not modify PP-MNPs absorption at this wavelength, and recovery was constant in the FFF analyses. Then, we considered the area percentage of HSA-PP-MNPs eluted at 8 min, obtaining the curve in Figure 8. To evaluate the binding trend we also increased the ratio between PP-MNPs and HSA to 1.6, 1:8, and 1:10, where population 2 was no longer visible and the only species was the core-shell structure already observed in population 1.

Until the HSA amount is double that of the PP-MNPs, there is very little interaction. This rapidly changes with a 1:2 ratio, indicating that only a higher amount of protein can interact with the PP-MNPs, with HSA encapsulation on PP-MNPs. When a 1:8 ratio is reached, all PP-MNPs are converted into HSA-PP-MNPs eluted as population 1.

Following a further HSA increase and PP-MNP dilution in the biological medium, until reaching the albumin range in healthy human blood (34–54 g/L), all PP-MNPs are coated with an HSA protein corona, acting as an HSA-PP-MNPs composite. The latter can be considered as biocompatible NPs due to the HSA shell, and suitable for potential therapeutic medical applications [57].

Some study limitations apply, such as the need for FFF method adjustment according to the type of sample, which requires a reoptimization if the methodology is applied to another system. The current FFF setup requires very little sample but is not indicated for particle collection and further study. If necessary, a scaling up to the flat, non-miniaturized channel can be performed. Limitations aside, combining these results with those obtained with batch techniques, it is evident how the information obtained is complementary and the approach is advantageous. Whereas DLS and Z potential evaluation allow an observation of the evolution of PP-MNPs when injected in a biological fluid, it is only through the selective size/conformation analysis obtained from FFF-multidetection that it is possible to visualize what species are present in a frame-by-frame approach, to observe the evolving PP-MNP surface, and to and understand the high excess of protein needed to achieve full conversion into their biological identity.

## 4. Conclusions

Understanding the evolution of MNP-based therapeutics upon injection into the patient is both necessary and challenging. It is crucial to identify possible particle instability that may raise safety concerns, but at the same time several parameters (size, conformation, coating, surface properties) need to be monitored in a dynamic way, which is difficult to achieve. While current techniques can monitor these parameters, their response is mediated upon all the possible populations present in the sample, since measurements are performed statically. A separation approach can instead provide selective information and identify particle evolution in the native state, monitoring the evolution of injected MNPs at increasing levels of protein and simulating their dispersion in a biological medium. In this work, a method based on miniaturized FFF-multidetection allowed us to confirm that the particles are relatively stable in the medium (though some aggregation can occur) and progressively interact with proteins to form a new biological identity, with a process that involves a very imbalanced mass ratio. By combining conformation studies to batch characterization results, it was also possible to confirm the unchanged morphology of particles which is strictly linked to their activity and cytotoxicity. With this approach and setup, it is possible to monitor nanoparticles–protein interactions in the very first steps of infusion and evaluate the effect of the concentration and ratio on the size, shape, and arrangement of PP-MNPs.

## Figures and Tables

**Table 1:** Experimental concentration setup for the DLS/ELS measurements.

PP-MNP:HSA Weight Ratio	PP-MNP		HSA	
	Volume (mL)	Concentration (mg L^−1^)	Volume (mL)	Concentration (mg L^−1^)
2:1	1	200	1	100
1:1		200		200
1:2		200		400
1:4		200		800

**Table 2:** Flow conditions for the HF5 analyses.

Focus (mL min^−1^)	Focus-Injection (mL min^−1^)	Elution (mL min^−1^)			Elution-Inject (mL min^−1^)
Vx = 0.8	Vx = 0.8	Vx = 0.55 to 0.04	Vx = 0.04	Vx = 0.00	Vx = 0.00
T = 1 min	T = 5 min	T = 6 min	T = 18 min	T = 3 min	T = 2 min

**Table 3:** Colloidal properties (hydrodynamic diameter, d_DLS_ and Z potential, Zpot_ELS_) of PP-MNPs and HSA.

Sample	Concentration (ppm)	dDLS (nm)	PDI	Z_pot_ (mV)	pH
PP-MNP	50	74 ± 1	0.1	−49.5 ± 3.3	7.3
	256	76 ± 2	0.2	−49.0 ± 2.4	7.4
HSA	800	9.8 ± 2.4	0.7	−31.5 ± 9.6	7.3
	100	10.4 ± 2.2	0.7	−33.5 ± 4.7	7.3

**Table 4:** DLS/ELS data for the titration of PP-MNPs with HSA.

PP-MNP/HSA	pH	Size d_DLS_ (nm)	Deviation (nm)	PDI	ζ-pot (mV)	Deviation (mV)
2	6.4	377	99	0.500	−40.2	0.7
1	6.7	263	53	0.450	−39.2	1.0
0.5	6.7	179	9	0.370	−41.9	0.5
0.25	6.6	187	4	0.400	−41.1	1.5

## Data Availability

The datasets generated for this study can be obtained from the corresponding author upon reasonable request.
